# Prognostic Utility of the Flow Cytometry and Clonality Analysis Results for Feline Lymphomas

**DOI:** 10.3390/vetsci11080331

**Published:** 2024-07-24

**Authors:** Sheena Kapoor, Sushmita Sen, Josephine Tsang, Qi-Jing Yap, Stanley Park, Jerry Cromarty, Deanna Swartzfager, Kevin Choy, Sungwon Lim, Jamin Koo, Ilona Holcomb

**Affiliations:** 1 ImpriMed, Inc., 1130 Independence Avenue, Mountain View, CA 94043, USA; sheena@imprimedicine.com (S.K.); sushmita.sen@imprimedicine.com (S.S.); jgtsang@imprimedicine.com (J.T.); qjyap@imprimedicine.com (Q.-J.Y.); stanleyp@imprimedicine.com (S.P.); jerrycromarty@imprimedicine.com (J.C.); dmswartzfager@gmail.com (D.S.); sungwon@imprimedicine.com (S.L.); 2 BluePearl Pet Hospital, Kirkland, WA 98034, USA; kevin.choy@bluepearlvet.com; 3 Department of Chemical Engineering, Hongik University, Seoul 04066, Republic of Korea

**Keywords:** feline lymphoma, flow cytometry, oncology, prognosis, clonality

## Abstract

**Simple Summary:**

We present flow cytometry and clonality evaluation as a reliable and useful method for characterizing feline lymphomas. Neoplastic cell size is a crucial factor for prognosis. We have developed a novel system for cell sizing by flow cytometry that was 82–90% concordant with the gold standard of cytology. Moreover, from our retrospective analysis of survival for small versus large cells, we saw consistency between both methodologies. These results highlight the utility of our approach in providing prognostic insights.

**Abstract:**

Feline lymphoma, a prevalent cancer in cats, exhibits varied prognoses influenced by anatomical site and cellular characteristics. In this study, we investigated the utility of flow cytometry and clonality analysis via PCR for antigen receptor rearrangement (PARR) with respect to characterizing the disease and predicting prognosis. For this purpose, we received fine needle aspirates and/or blood from 438 feline patients, which were subjected to flow cytometry analysis and PARR. We used a subset of the results from patients with confirmed B- or T-cell lymphomas for comparison to cytological or histological evaluation (n = 53). Using them as a training set, we identified the optimal set of flow cytometry parameters, namely forward scatter thresholds, for cell size categorization by correlating with cytology-defined sizes. Concordance with cytological sizing among this training set was 82%. Furthermore, 90% concordance was observed when the proposed cell sizing was tested on an independent test set (n = 24), underscoring the reliability of the proposed approach. Additionally, lymphoma subtypes defined by flow cytometry and PARR demonstrated significant survival differences, validating the prognostic utility of these methods. The proposed methodology achieves high concordance with cytological evaluations and provides an additional tool for the characterization and management of feline lymphoproliferative diseases.

## 1. Introduction

Feline lymphoma is one of the most prevalent forms of neoplastic disorder in cats, accounting for nearly 30% of all feline cancers [1,2]. The incidence rate varies between 30 and 200 cases per 100,000 cats and presents across diverse anatomical sites. Alimentary lymphoma is the most frequent presentation, followed by mediastinal and multicentric lymphoma [3,4,5,6,7,8].

The diverse clinical behaviors and outcomes observed in various subtypes of feline lymphoma emphasize the pivotal need for characterization and prognostication. For example, the alimentary form of lymphoma can manifest as either small or large cells, which are generally considered indolent or aggressive, respectively [9]. Indeed, the size of neoplastic cells for feline lymphoma, for most anatomical forms, has been shown to have a significant impact on outcomes [9,10,11,12,13]. Small-cell lymphoma offers a more favorable prognosis with longer survival and milder symptoms. Conversely, cats with large-cell lymphomas show significantly shorter survival. High-grade lymphoma, which consists mostly of large or intermediate cells, is generally treated with more aggressive chemotherapy regimens such as CHOP [6,14,15]. Low-grade lymphomas, which are characterized by small cells, can respond favorably to less intensive single agent protocols such as lomustine and chlorambucil with or without a corticosteroid taper [9,14]. Identifying precise cell sizes in the context of immunophenotype and clonality might better support the choice of effective treatment strategies and predict patient prognosis.

Currently, cytological or histopathological examinations, typically carried out via fine-needle aspiration cytology (FNAC), are considered the gold standard for diagnosing lymphoproliferative diseases and providing cell-size evaluation. However, these methods require a pathologist, with high-level training and years of expertise, who can discriminate between reactive and tumor-derived lymphoid cells to provide a reliable determination of the cell size for the latter. Cytological methods requiring an expert of this level are time-consuming and can be prone to individual interpretation.

The potential of flow cytometry (FC) in sizing cells and immunophenotyping in feline lymphoma, to date, is relatively unexplored. Guzera et al. showcased the utility of FC in distinguishing between neoplastic and non-neoplastic lymphocyte populations and immunophenotyping lymphoproliferative disorders in cats [16]. More recent reports have further shown the utility of FC but with a limited number of antigenic targets or with small sample sizes. Martini et al. examined immunophenotyping in cats by expression patterns of two targets, CD44 and CD18, in feline leukocytes [17]. Another recent report used FC to set reference intervals for reactive versus normal lymph nodes for 24 of 31 cats [18]. While these studies show promising capabilities for FC in the field of feline lymphoma, more extensive research is warranted.

In conjunction with clonality testing, we propose a flow-cytometry-based method as a powerful technique for feline lymphoma characterization. While it is already utilized in human and canine medicine for identifying and characterizing lymphoma, there is a noticeable absence of its utilization in feline veterinary medicine [19,20,21,22,23]. In this study, we conducted flow cytometry and clonality analysis using isolated cells derived from feline patients. The results were compared to the cytological or histological assessment of cell size to understand the relationship between the two and develop the sizing scheme based on flow cytometry analysis. The clinical outcomes of the cell size groups based on flow cytometry and/or clonality are shown to illustrate the prognostic utility of the proposed approach for characterizing feline lymphomas.

## 2. Materials and Methods

### 2.1. Patient Population

All feline patient specimens were collected under informed consent, and the experiments were approved by the internal review board, including the ethics committee (IMVLSA0614.20). Between July 2020 and December 2023, ImpriMed, Inc. (Mountain View, CA, USA) received 438 feline patients’ samples. The specimen submission consisted of 337 fine needle aspirates (FNA), 294 whole blood samples, and 48 cytology slides. The FNAs and whole blood were collected using 20 or 22 G needles in proprietary ImpriMed transport media as previously described by Bohannan et al. [24]. The samples were then shipped overnight and processed within 24–48 h of collection. Patient data, including but not limited to age, gender, breed, anatomic sampling site, cytological evaluation and immunophenotype (if known), diagnosis, and treatment, were recorded for analysis.

### 2.2. Flow Cytometry Analysis

Flow cytometry was performed on lymphoid cells isolated from FNA and/or whole blood samples in the previously described manner [24]. Peripheral blood mononuclear cells (PBMCs) were extracted from whole blood following the manufacturer’s instructions (StemCell Technologies, Vancouver, BC, Canada). A minimum of 4.0 × 10^5^ cells were required to perform the following analysis. The isolated lymphocytes were stained with different combinations of directly conjugated antibodies consisting of Fluorescein Isothiocyanate (FITC), Phycoerythrin (PE), Alexa Fluor 647 (AF647) and Allophycocyanin (APC), respectively, to analyze different antigen expressions to determine the immunophenotype. Antibodies included anti-CD4 (Clone vpg34) and anti-CD8 (Clone vpg9) for identifying helper and cytotoxic subsets of T lymphocytes, respectively. Anti-CD21 (Clone CA2.1D6) was used to characterize B-cell lymphomas. Anti-CD5 (Clone YKIX322.3) and anti-CD14 (Clone TUK4) were used to identify T-cells and monocytes, respectively. Additionally, anti-CD18 (Clone CA1.4E9) was used to determine the proportion of leukocytes in the sample. This antibody combination provided a comprehensive cellular profile of the samples (Table S1). When a predominant population of either B- or T-cells was not identified in a sample using flow cytometry, the results were categorized as “Not-Determined” (ND).

The cells were stained with antigen-specific antibodies and isotype controls at a concentration of 2 to 10 μg/mL in phosphate-buffered saline (PBS) with 1.0% bovine serum albumin (BSA) for 30 min in the dark at 4 °C, and then washed using manufacturer recommended protocols on the HT1000 Curiox Biosystem (Woburn, MA, USA). Ten thousand cells were acquired from each reaction. All flow cytometry was performed with a Guava easyCyte HT8 Flow System (Luminex, Austin, TX, USA) and data analysis was carried out with FCS Express 6 De Novo software (Pasadena, CA, USA). Cell population was gated based on the linear forward scatter (FSC) versus side scatter (SSC) plots, after the exclusion of dead cells and granulocytes (Figure S1). The average lymphocyte population was 69%, ranging from 31% to 96% across the samples. When the antigen fluorescence was higher than that of an antibody isotype control, the antigen expression was considered positive (Figure S2).

### 2.3. Training of Cell Size Distinction Based on Flow Cytometry

We included only feline patients that met the following criteria for calibrating cell sizing windows: (1) a suspected lymphoma diagnosis by a general practitioner veterinarian or veterinary oncologist; (2) a cytology report confirming neoplasia with a description of cell morphology and size by a pathologist; and (3) concordant determination of either B- or T-cell lymphoma by both flow cytometry and PARR. The 53 samples meeting the above criteria were used as a training set, and they were grouped by the size of the neoplastic cells as determined by a veterinary pathologist based on cytology. We sought to classify the isolated cells as having a “small”, “intermediate”, or “large” size based on the median FSC values computed from the flow cytometry analysis. Optimization of the threshold FSC values for the size classification proceeded under the following scheme:

$$\max\;concordance\;score=\frac{1}{48.75}(1.00\;n_{c}+0.75\;n_{s})$$

(1) $$s.t.\;\;n_{c}+n_{s}+n_{i}=53;\;$$

(2) $$\;n_{c},\;n_{s},\;n_{i}\geq 0$$

(3) $$n_{c}=\sum_{k=1}^{53}\delta \left(f_{k},c_{k}\right);$$

(4) $$n_{s}=\sum_{k=1}^{53}1[(f_{k}\cap c_{k}\neq \emptyset )\wedge \;{(f}_{k}\neq c_{k})]$$

(5) $$f_{k}=\{small,\;medium,\;large\}\;\mathrm{where}\;{FSC}_{k}\leq z_{s}\;⟹\;f_{k}\;=\;”\mathrm{small}”$$

$${FSC}_{k}\geq z_{l}\;⟹\;f_{k}\;=”\mathrm{large},\;z_{s}<{FSC}_{k}<z_{l}\;⟹\;f_{k}\;=\;”\mathrm{intermediate}”$$

(6) $$\;c_{k}=\{small,\;\{small,\;intermediate\},\;intermediate,\;\{intermediate,\;large\},\;large)$$

where $n_{c}$, $n_{s}$, and $n_{i}$ represent the number of cases for which the median FSC-based classification is congruent, semi-congruent, or incongruent in relation to the cytological assessment. $f_{k}$ and $c_{k}$ represent the cell size of the sample from the *k*th patient based on flow cytometry and cytology, respectively. $z_{s}$ and $z_{l}$ stand for the threshold median FSC values for small and large cell subtype, respectively. Cytological assessments of cell size “small to intermediate” and “intermediate to large” are defined as {small, intermediate} and {intermediate, large}, respectively. The concordance score defined in the above will be computed as follows. If the sizing matched the determination of size by median FSC exactly, the sample will be assigned a score of “1.00”. If the sizing overlapped with the sizing by median FSC (e.g., “small to intermediate” based on cytology and “small” based on flow cytometry), the sample will be assigned a score of “0.75”. If there is no consistency in the sizing calls, the sample will be assigned a score of “0.00”. The score is calibrated to account for the number of “small to intermediate” and “intermediate to large” in the dataset, which is 17, such that it has a maximum of 1.00 and minimum of 0.00.

### 2.4. Validation of the Cell Sizing

Separate from the 53 samples in the training set, 24 patients were used to validate the FSC-based cell sizing. The 24 patients were withheld and only used after the FSC-based sizing thresholds were chosen using the training set. These patients were selected randomly in a stratified manner to preserve the proportion of the different cell size groups in the training cohort. Of the 24, 16 patients had the cytology results, including cell-size determination, in the medical charts. For the remaining eight, we prepared cytological smears from the same sample of cells used for flow cytometry analysis. Cytological analysis of these samples was performed by Veterinary Diagnostics (Davis, CA, USA). The resultant cell size classifications were compared to the median FSC-based sizes to calculate concordance score as defined in Section 2.3.

### 2.5. PCR for Antigen Receptor Rearrangements (PARR)

We utilized PARR to detect clonal populations, conforming to the protocols described previously in Rout et al. [25]. In brief, genomic DNA (gDNA) was extracted from feline lymph nodes or whole blood samples, which had at least 2 × 10^5^ cells, using QIAmp DNA Minikit following the manufacturer’s instructions (Qiagen, Hilden, Germany). Each sample was subjected to PCR using the previously reported set of primers [25]. Each PCR reaction included positive controls (KO-1 and MS4 cell lines) and non-template controls. Clonality was assessed following the criteria outlined by Waugh et al. [26], where distinct peaks identified clonal samples, and a Gaussian or skewed distribution indicated polyclonal samples. Samples were categorized as B- or T-cell clonal based on the specific primer set [25] ranges. Those showing polyclonal patterns or no amplification with both primer sets were considered negative for clonality [26,27].

### 2.6. Survival and Statistical Analysis

The index date was the date of diagnosis. Progression-free survival (PFS) was measured from the index date to the date of disease progression or death (including euthanasia), while overall survival (OS) was calculated from the index date to only the latter. Survival analysis was performed using the Kaplan–Meier method. Differences in survival were tested using the log-rank test. Hazard ratios were calculated using the Cox proportional hazards model. The significance level was set at *p* < 0.05. Analyses were performed using Python (version 3.9.7) or Prism 8 (GraphPad, San Diego, CA, USA).

## 3. Results

### 3.1. Case Selection

Due to low cellularity from FNA and/or whole blood, 204 patients were excluded from this study. This resulted in 234 cats with both flow cytometry and PARR results. Veterinarians were periodically contacted at three- or six-month intervals for patient medical charts after the flow cytometry and PARR results were provided. Of these 234 patients, we received the corresponding medical charts with outcome data for a total of 126 patients. Baseline clinical characteristics of these remaining group are provided in Table 1.

The majority of feline patients that met our inclusion criteria were diagnosed with alimentary lymphoma. FNA samples were taken from various sites, with mesenteric and submandibular lymph nodes being the most frequent. The most common breed was domestic shorthair (65%), followed by longhair (10%). The median follow-up was 6 months.

### 3.2. Comparison between Cytological Cell Size and Flow Cytometry Results

We identified 53 cases that met the criteria for inclusion in defining cell sizing windows using the median FSC values from flow cytometry analysis. The details of these patients are given in Table S2. In summary, 36 and 17 of the cases represented T- and B-cell lymphomas, respectively (Table 2). By cytology, the size categories represented were small, small to intermediate, intermediate, intermediate to large, and large. Furthermore, 56% of the T-cell cases were predominantly labeled small or small-to-intermediate, with the rest being similarly distributed amongst the remaining categories. In contrast, the B-cell lymphomas were mostly (88%) classified as intermediate to large or large by cytology.

The FSC of the flow cytometry results is proportional to the size of particle, i.e., the cell in our experiments. We thus compared the distribution of FSC values across the five different cell sizes determined by cytology. We observed the expected proportionality between the two except for the small-to-intermediate class, which had a notably lower median FSC that the small class does. Given these results, we determined the non-overlapping range of median FSC values for the three cell sizes—small, intermediate, and large—that minimizes the incongruence between the size dictated by cytology and that by flow cytometry analysis. Using the threshold values listed in Table 2, the concordance rate was 82% and we observed five cases where the FSC-based size did not agree with the cytological assessment. When we tested this FSC-based sizing scheme on the independent, test dataset of 24 additional patients, concordance with the cytological and/or histological evaluation was 90% (Table 3). Notably, the intermediate- and intermediate-to-large-sized cells were underrepresented in this dataset. Only one of those cases (F0254), for which the immunophenotype was not determined, showed a completely non-overlapping size category between the cytology and FC-based size—small vs. large. For the other five cases, our FC analysis suggested a distinct size category, whereas the pathological review showed a range.

### 3.3. Prognostic Utility of the Flow Cytometry Results and PARR

**Figure 1:**
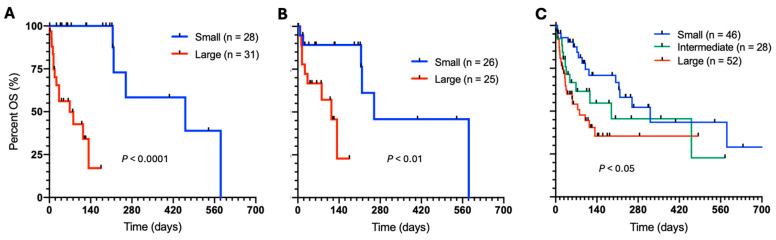
Survival of the feline lymphomas with respect to cell size. Small vs. large subtype based on (**A**) cytology, and (**B**) flow cytometry results. There were 8 and 16 patients with intermediate cell size in (**A**) and (**B**), respectively. (**C**) Comparison of the small, intermediate, and large subtype based on the flow cytometry results. The scale and label of the *y*-axis are the same from (**A**–**C**).

We compared the survival outcomes of the naïve feline lymphoma patients (n = 67) stratified according to cell size based on cytology (Figure 1A) or FC analysis (Figure 1B). These 67 patients are a subset of the 77 patients in the combined training and validation set used for FSC calibration. This group of patients had a robust diagnosis along with paired cytological and FC analysis results, which allows for comparison with respect to prognostic utility. Only the patients marked as having a small or large cell size were considered, leading to 59 vs. 51 patients for cytology vs. FSC-based cell size comparison, respectively. The results demonstrated a significantly superior OS of the naïve patients diagnosed with small-cell lymphomas than the group with large cell subtype based on cytology (Figure 1A). The hazard ratio (HR) was 6.3 (95% CI: 2.4–16.6). The difference in survival was also significant when comparing the two cell size groups based on the flow cytometry results (Figure 1B). HR of 3.4 (95% CI: 1.1–10.1) was observed. We observed that the significant difference in survival between small and large cell size subtypes remained to be true (*p* = 0.0045) when the analysis was carried out with both the naïve (n = 67) and relapsed (n = 10) patients (Figure S3).

After expanding the inclusion criteria to patients with clinical information along with FSC-based cell sizes, we analyzed the survival of the entire cohort (N = 126). A significant difference in OS was observed between the small-, intermediate-, and large-cell lymphomas (Figure 1C). The pairwise *p* values between three groups—small vs. intermediate, small vs. large, and intermediate vs. large—were 0.09, 0.0045, and 0.32, respectively. Notably, the median OS of the patients with the intermediate cell size was 189 days, positioned between 321 and 81 days for the small- and large-cell subtypes, respectively.

**Figure 2:**
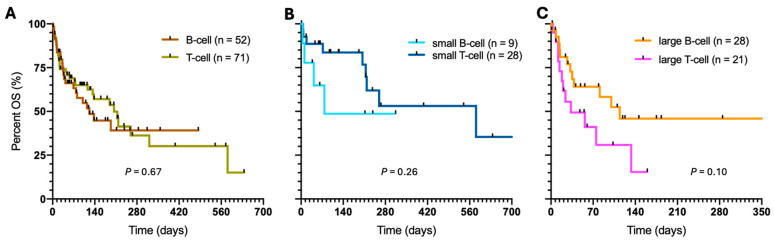
Survival of the feline lymphomas with respect to clonality and cell size. (**A**) OS of the B- vs. T-cell clonal subtype. Influence of clonality on the OS of (**B**) small- and (**C**) large-cell lymphomas. The scale and label of the *y*-axis are the same from (**A**–**C**).

Next, we compared the survival of B- vs. T-cell clonal feline lymphomas within the entire cohort with clinical outcome (N = 126), excluding three patients with not-determined immunophenotype. The median OS of the two clonalities based on PARR was 122 and 204 days, respectively (Figure 2A). However, the difference was not statistically significant (*p* = 0.67). When combined with the cell size based on median FSC, a tendency of superior survival among the small, T-cell lymphomas than the small, B-cell subtype was observed without statistical significance (Figure 2B). Notably, only 24% of the small-cell lymphomas had B-cell clonality, which may have contributed to insufficient statistical power of the survival comparison. The survival of the small vs. large B-cell lymphomas was not significantly different (*p* = 0.95). Similarly, the OS of the large-cell lymphomas with B-cell clonality tended to be superior to the survival of the large cell with T-cell clonality (Figure 2C). In addition, 57% of the large cell had B-cell clonality.

### 3.4. Influence of Operational Factors on the Analysis Results

In order to understand how inclusion criteria and the nature of samples received affected the results, we tried analyzing the results of the samples under more strict terms as follows: (1) only FNA samples and comparison to cytology; and (2) only alimentary forms that meet the first criterion (FNA and cytology). When adopting the former inclusion criterion, 63 cases out of 126 remained. Applying the latter inclusion criterion resulted in 26 cases. The concordance rate with respect to cell size and hazard ratios between the small and large cell were different from the values obtained with the entire cohort, as shown in Table 4. The concordance rate with respect to cell size based on cytology vs. FSC was much higher when the analysis was based on FNA (88%) than blood (71%). We also noted that when the cytology was restricted to standard cytology (excluding the cytospin-based ones), the concordance rate of the group (N = 44) improved by 0.6%. Limiting the lymphomas to the alimentary forms analyzed using FNA resulted in a significant increase (9%) in the concordance rate. The OS of the large cell groups remained significantly inferior to the survival of the small cell groups, as determined by FSC, regardless of the changes in the inclusion criteria.

## 4. Discussion

Once anatomic form is determined or suspected clinically, key features in the accurate characterization of and prognostication for feline lymphoma rely on cellular and subcellular factors. The most important features include neoplastic cell size and grade, of which the latter is determined based on morphology and cellular composition of specimen [2,6,28]. With the histological analysis being the golden standard, cytology has also been employed to obtain relevant information. In this study, we characterized the isolated cells derived from FNA and/or whole blood of feline lymphoma patients via flow cytometry and PARR. The results demonstrate that the cell size and clonality based on these rapid, less invasive methods can also provide useful insights on prognosis of the patients.

We observed an overall concordance rate of 86% between flow-cytometry-based cell sizing and cytological or histological assessment. In the validation set, only one case exhibited a completely different resultant size. We suspect that incongruence was due to the sample (blood with inconclusive immunophenotype) not being able to fully represent the patient’s cancer. The hazard ratios of the resultant cell size groups were also similarly high with markedly different survival, highlighting a robust agreement between these diagnostic modalities. Flow cytometry allows for the analysis of tens of thousands of cells, potentially including a mix of neoplastic and non-neoplastic cells, providing a broad overview of the cellular environment. In contrast, cytological and histological assessments typically involve the examination of a few hundred cells that are stained to enhance the visualization of morphological characteristics, enabling neoplasm identification. These methodological differences may account for the subtle variances observed in cell size measurements and their resultant impact on clinical outcomes. While flow cytometry offers a more comprehensive quantitative analysis, cytology and histology provide a focused qualitative assessment, each contributing uniquely to the overall diagnostic and prognostic framework.

Our study was collaboratively conducted across multiple veterinary hospitals and with various oncologists (see Acknowledgements), utilizing datasets derived from real-world clinical settings. This approach lends practical relevance to our findings but also introduces limitations and potential variability due to differing clinical practices among participating institutions and veterinarians. In this regard, it is worthwhile to note that we achieved a higher concordance rate when compared to the cytological results conducted by the single institution (Table 3) than the comparison to the results obtained from multiple institutions. These results suggest that cytological interpretation may be different across operators and/or institutions. We also observed an improved concordance rate when the analysis is limited to the samples with standard cytology and FC performed on FNA. This observation hints at the potential variability in cell size among different techniques and/or samples—FNA vs. PBMC, cytology vs. histology, and/or standard cytology vs. cytospin from resuspended FNA.

The results of our study align closely with existing literary reports, corroborating the clinical utility of our findings. Specifically, we observed that large-cell lymphomas are associated with poorer OS (median, 2 months) compared to small-cell subtypes (median, 15 months), which is consistent with the literature [2,6,14,28]. Small-T-cell lymphomas exhibited superior survival with median OS of 19 months, whereas large-T-cell lymphomas faced the shortest median OS of 1 month. These results are in concordance with previously published reports [7,9,12,13,14,23] and underscores the prognostic value of employing flow cytometry and PARR for sizing and determining the subtype of the lymphoma. Overall, the insights gained through our research highlight significant prognostic distinctions within lymphoma subtypes, reinforcing the need for precise diagnostic techniques in veterinary oncology.

It is important to note that the failure rate of the FNA samples received was notably larger for feline lymphomas than canine lymphomas in our experience [24]. This may have been due to employing a needle gauge of 20 or 22 G for FNA, which may be too thick for cats. A recent study suggested a 21 G needle to be optimal for feline patients [29]. The high rate of unsuitable feline samples for flow cytometry analysis has been reported by others as well [29]. We were not able to conduct both flow cytometry and PARR for 36% of the patients. When limited to flow cytometry, the failure rate was 23%. This is comparable to the rates reported in the literature [16,29]. The majority of the failures were due to an insufficient number of lymphocytes in the sample. This compares unfavorably to only 13% of the samples derived from canine lymphomas. An encouraging trend was noted in the last 100 samples, where the failure rate decreased to approximately 27%, indicating that improved sampling techniques or greater experiential knowledge could mitigate issues of low cellularity. However, the persistence of a relatively high failure rate suggests that a myriad of factors, including the challenge of obtaining adequate samples from anatomical sites like the abdomen and the behavioral stress response in felines during biopsy procedures, contribute to these difficulties. Ongoing improvements in both the devices used for sampling and the methodology itself are essential for the broader adoption of our proposed diagnostic approach for feline lymphomas. In this regard, the improvements seen in next-generation flow cytometry [30] are encouraging and may help to resolve some of the aforementioned limitations.

## 5. Conclusions

Our results show that the combined use of flow cytometry and PARR on FNA and/or blood samples can provide useful insights for feline lymphomas. The identification of cell size and clonality based on these methods achieved comparable prognostic utility. These methods also provide information about antigen expression profile of the cells in the affected site that may help refine subtypes of the disease. Furthermore, the application of flow cytometry and PARR as complementary diagnostic tools holds promise for refining the clinical workflow, enabling a more rapid and non-invasive assessment of feline lymphomas. Future work will focus on enhancing these technologies to reduce sample failure rates and improve prognostic accuracy, ultimately leading to tailored therapeutic strategies and better clinical outcomes for feline patients.

## Figures and Tables

**Table 1:** Clinical characteristics of the cohort of 126 available medical records.

Characteristic	Overall (%)
Age (years)	
Median (range)	11 (1–21)
Sex	
Male	58
Female	41
Not indicated	1
Anatomic site	
Alimentary	42
Multicentric	27
Other	26
Not indicated	5
Cell size ^a^	
Small to intermediate	40
Intermediate to large	52
Not indicated	8
Naïve vs. relapse	
Naïve	79
Refractory/relapse	17
Not indicated	4

^a^ This is based on the medical chart provided by the veterinarians.

**Table 2:** Cytological cell sizes and flow cytometry results of the 53 patients.

Cell Size by Cytology	Number	Median FSC (Median ± Stdev, a.u.)	Calibration Standard in Terms of FSC
Small	16	230 ± 6	≤236
Small to Intermediate	5	219 ± 7	
Intermediate	5	240 ± 5	>236 and <272
Intermediate to Large	12	284 ± 12	≥272
Large	15	291 ± 7	

abbreviations: FSC, forward scatter; a.u., arbitrary unit.

**Table 3:** Concordance using the independent test dataset (n = 24).

Sample ID & Type	Immunophenotype ^b^	Cytology-Based Size	Median FSC (a.u.)	FSC-Based Size	Concordance Score
F0101, FNA	ND	Small	209	Small	1.00
F0104, FNA	B-cell	Small to intermediate	223	Small	0.75
F0123, FNA	B-cell	Small	201	Small	1.00
F0129, FNA	B-cell	Small to intermediate	258	Intermediate	0.75
F0140, FNA	T-cell	Intermediate	247	Intermediate	1.00
F0158, FNA	ND	Large	282	Large	1.00
F0165, FNA	B-cell	Large	313	Large	1.00
F0174, FNA	B-cell	Small	199	Small	1.00
F0185, FNA	ND	Small	219	Small	1.00
F0202, FNA	B-cell	Large	375	Large	1.00
F0203, FNA	ND	Small	214	Small	1.00
F0210, FNA	T-cell	Large	363	Large	1.00
F0231, FNA	T-cell	Large	299	Large	1.00
F0237, FNA	B-cell	Small	203	Small	1.00
F0239, FNA ^a^	T-cell	Small	199	Small	1.00
F0254, Blood	ND	Small	292	Large	0.00
F0288, FNA ^c^	T-cell	Intermediate to large	303	Large	0.75
F0346, FNA ^c^	B-cell	Large	291	Large	1.00
F0396, Blood ^c^	T-cell	Small to intermediate	245	Intermediate	0.75
F0452, FNA ^c^	T-cell	Small	212	Small	1.00
F0454, FNA ^c^	T-cell	Small to intermediate	271	Intermediate	0.75
F0457, Blood ^c^	T-cell	Small to intermediate	226	Small	0.75
F0459, FNA ^c^	T-cell	Small	225	Small	1.00
F0462, FNA ^c^	T-cell	Small	194	Small	1.00

^a^ We received and analyzed both FNA and blood sample for the patient F0239. ^b^ ND: Not determined via flow cytometry analysis (see Section 2). ^c^ Cytology reviewed by the single institution as indicated in Section 2.

**Table 4:** Comparison of the concordance rate and hazard ratios across the cohorts with varying inclusion criteria.

	Cytology ^a^	FNA and Cytology ^b^	Alimentary Forms ^c^
Number of cases	77	50	16
Concordance rate	82%	88%	91%
Hazard ratio ^d^	3.7	4.3	2.8

^a^ The results of the cohort with both cytology and flow cytometry are included. ^b^ The results of the cohort with both cytology and flow cytometry based on FNA are included. ^c^ The results of the cohort with both cytology and flow cytometry based on FNA and suffering from alimentary forms of feline lymphoma are included. ^d^ The hazard ratio of overall survival between the large and small cell size group based on the flow cytometry is shown.

## Data Availability

Data contained within this article and Supplementary Material.

## Supplementary Materials

The following supporting information can be downloaded at: https://www.mdpi.com/article/10.3390/vetsci11080331/s1, Figure S1: Gating of the targeted cells during the flow cytometry analysis; Figure S2: The histogram of fluorescence from the antibody (Anti-CD5, blue) versus negative control (red) labeled cells; Figure S3: Survival of the feline lymphomas with respect to cell size; Table S1: List of the antibodies used to label the cells and analyze antigen expression; Table S2: List of the patients included in the training cohort.
