# Supplementary material for: Prognostic Utility of the Flow Cytometry and Clonality Analysis Results for Feline Lymphomas
Source: Vet Sci. 2024 Jul 24;11(8):331. doi: 10.3390/vetsci11080331 (PMC11360694; doi:10.3390/vetsci11080331)
Supplement: Supplementary file 1 [file vetsci-11-00331-s001.zip › vetsci-3008933-supplementary.pdf]

Supporting Information

Prognostic utility of the flow cytometry and clonality analysis results for feline lymphomas

Sheena Kapoor[1,+], Sushmita Sen[1,+], Josephine Tsang[1], Qi Jing Yap[1], Stanley Park[1], Jerry Cromarty[1], Deanna Swartzfager[1], Kevin Choy[2], Sungwon Lim[1], Jamin Koo[1,3,*] and Ilona Holcomb[1,*]

[1]ImpriMed, Inc., 1130 Independence Avenue, Mountain View, CA 94043, United States
[2]BluePearl Pet Hospital, Kirkland, WA 98034, USA
[3]Department of Chemical Engineering, Hongik University, Seoul 04066, Republic of Korea

[+]These authors contributed equally to this work.
***Correspondence**: jaminkoo@alumni.stanford.edu and iholcomb@imprimedicine.com

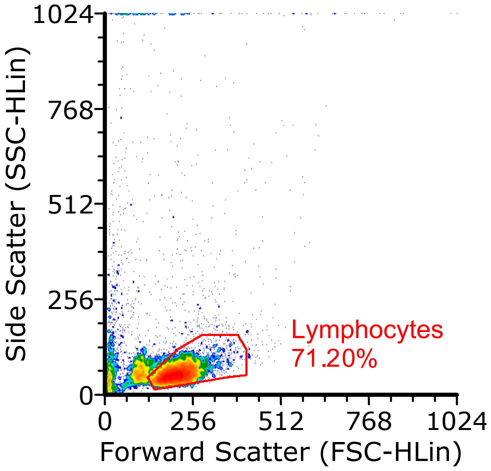

Figure S1. Gating of the targeted cells during the flow cytometry analysis. Forward scatter (FSC) and side scatter (SSC) plots are used to gate the lymphocytes, which are enclosed within the red lined box in the plot shown in the above. This gating is based on the previous work [1-3] and proceeded as follows. The lymphocyte gate was originally defined from our initial research for canines but refined for feline specimens as follows. The FSC/SSC characteristics of feline derived lymphocytes was evaluated in normal lymph nodes (n=6) and paired blood specimens provided with suspected lymphoma patients with early stage disease (n=12). The gating definition was adjusted for the feline cells. This gating strategy excludes debris, dead cells, and non-lymphoid cells such as granulocytes and monocytes.

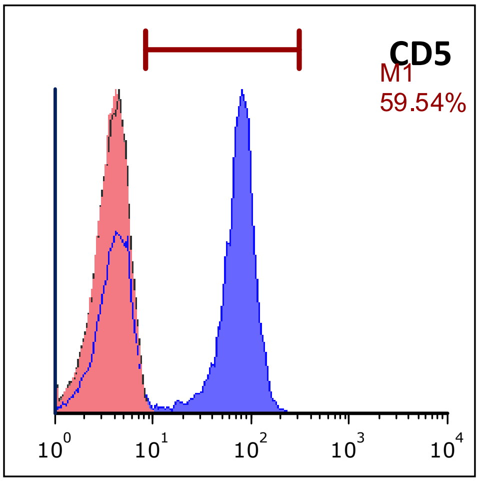

Figure S2. The histogram of fluorescence from the antibody (Anti-CD5, blue) versus negative control (red) labeled cells. The proportion of the antibody-labeled cells that exhibited fluorescence greater than the threshold level determined by the control (red)—59.54% —is indicated on the top right corner.

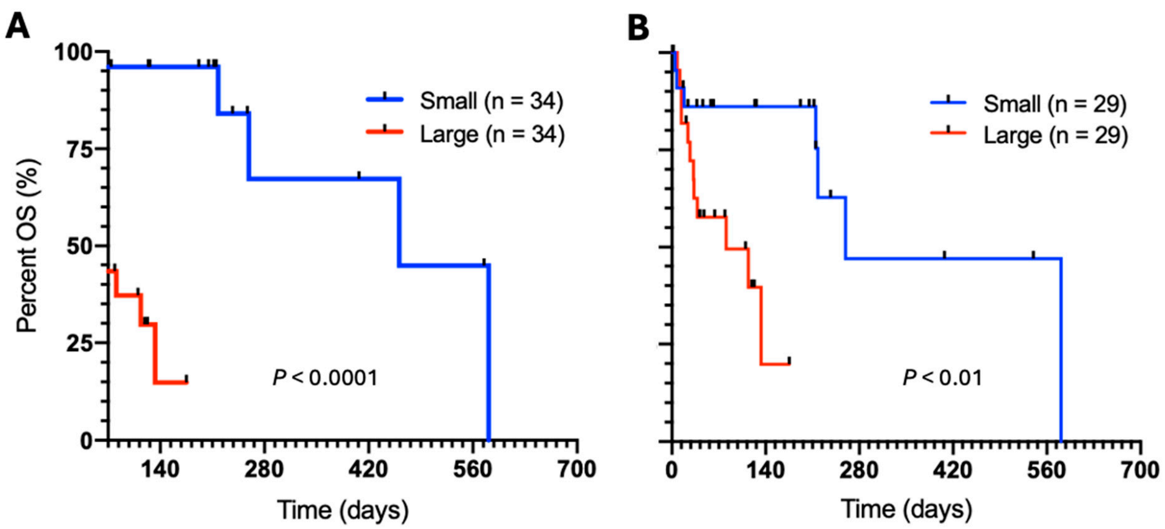

Figure S3. Survival of the feline lymphomas with respect to cell size. Small vs large subtype based on (**A**) cytology, and (**B**) flow cytometry results. Both naïve and relapsed patients were included in the analyses.

Table S1. List of the antibodies used to label the cells and analyze antigen expression.

Antibody	Clone	Isotype
CD4	vpg34	Mouse IgG1
CD4 control	-	Rat IgG2a
CD5	FE1.1B11	Mouse IgG1
CD5 control	-	Mouse IgG1
CD8	vpg9	Mouse IgG1
CD8 control	-	Rat IgG1
CD14	TUK4	Mouse IgG2a
CD14 control	-	Mouse IgG2a
CD18	CA1.4E9	Mouse IgG1
CD18 control	HM57	Mouse IgG1
CD21	CA2.1D6	Mouse IgG1
CD21 control	-	Mouse IgG1

Table S2. List of the patients included in the training cohort.

Sample ID	Age	Sex	Breed	Sample type	Immunophenotype
F0005	8years	FS	DSH	FNA	T
F0007	13 years 1 month	MN	Mixed	Blood	T
F0010	13 years 11 months	MN	Mixed	FNA	T
F0012	11 years	MN	Mixed	FNA	T
F0015	17 years	MN	Mixed	FNA	B
F0021	17 years	MN	Mixed	FNA	B
F0025	12 years 5 months	MN	Mixed	FNA	B
F0028	12 years	MN	Mixed	Blood	T
F0032	14 years	FS	Mixed	FNA	B
F0034	11 years 1 month	FS	Mixed	FNA	T
F0035	12 years 1 month	FS	Mixed	Blood	T
F0036	13 years 1 month	MN	Mixed	Blood	T
F0046	16 years 5 months	MN	Mixed	FNA	B
F0048	13 years	FS	Mixed	FNA	B
F0060	15 years	MN	Mixed	FNA	T
F0063	13 years	MN	DSH	FNA	B
F0075	8 years	FS	DSH	Blood	T
F0081	7 years 1 month	FS	DSH	Blood	T
F0087	12 years 1 month	MN	Ocicat	Blood	T
F0095	16 years 1 months	MN	DLH	Blood	T
F0098	12 years 9 months	FS	DSH	FNA	T
F0100	10 years	MN	DSH	Blood	T
F0108	10 years	FS	DSH	FNA	B
F0114	1 year 6 months	FS	DLH	FNA	T
F0116	13 years	MN	DSH	FNA	T
F0118	15 years 3 months	FS	DSH	Blood	T
F0120	18 years	MN	DLH	Blood	T
F0126	14 years	FS	DSH	Blood	T
F0127	10 years 11 months	FS	DSH	Blood	T
F0128	7 years 11 months	MN	DSH	FNA	T
F0137	12 years	MN	DSH	FNA	B
F0144	6 years	MN	DSH	Blood	T
F0146	20 years 2 months	FS	DLH	Unknown	T
F0149	9 years 7 months	FS	DSH	FNA	B
F0150	14 years	FS	DSH	FNA	B
F0159	12 years 3 months	FS	DSH	FNA	T
F0172	13 years	MN	DSH	Blood	B
F0177	14 years 10 months	FS	DSH	Blood	T
F0195	9 years 8 months	MN	Bobtail	FNA	T
F0205	7 years	FS	DSH	Blood	T
F0212	10 years 5months	MN	DSH	FNA	T
F0218	9 years 6 months	MN	DSH	FNA	T
F0225	13 years 4 months	MN	DSH	Blood	T
F0265	15 years 1 month	FS	DSH	FNA	T
F0274	13 years 8 months	FS	DSH	FNA	B
F0275	11 years 10 months	MN	DSH	Blood	T
F0285	12 years 6 months	FS	DSH	FNA	T
F0293	11 years 0 months	MN	DSL	FNA	B
F0322	5 years 5 months	MN	DSH	FNA	B
F0327	10 years 1 month	FS	DSL	FNA	B
F0330	13 years 1 month	FS	DSH	FNA	T
F0337	9 years 1 month	FS	DSH	FNA	B

Supplementary References

1. Bohannan, Zach, et al. "Predicting likelihood of in vivo chemotherapy response in canine lymphoma using ex vivo drug sensitivity and immunophenotyping data in a machine learning model." *Veterinary and Comparative Oncology* 19.1 (2021): 160-171.

2. Rout, Emily D., et al. "Assessment of immunoglobulin heavy chain, immunoglobulin light chain, and T-cell receptor clonality testing in the diagnosis of feline lymphoid neoplasia." *Veterinary clinical pathology* 48 (2019): 45-58.

3. Guzera, M., et al. "The use of flow cytometry for immunophenotyping lymphoproliferative disorders in cats: a retrospective study of 19 cases." *Veterinary and Comparative Oncology* 14 (2016): 40-51.
